# Mutual Influence of Reward Anticipation and Emotion on Brain Activity during Memory Retrieval

**DOI:** 10.3389/fpsyg.2017.01873

**Published:** 2017-10-25

**Authors:** Chunping Yan, Fang Liu, Yunyun Li, Qin Zhang, Lixia Cui

**Affiliations:** ^1^Learning and Cognition Key Laboratory of Beijing, College of Psychology, Capital Normal University, Beijing, China; ^2^College of Psychology, Xinxiang Medical University, Xinxiang, China

**Keywords:** monetary reward, emotion, retrieval, episodic memory, ERP

## Abstract

Previous studies on the joint effect of reward motivation and emotion on memory retrieval have obtained inconsistent results. Furthermore, whether and how any such joint effect might vary over time remains unclear too. Accordingly, using the event-related potential (ERP) measurement of high temporal resolution, our study investigates the cognitive and brain mechanisms of monetary reward and emotion affecting the retrieval processes of episodic memory. Twenty undergraduate and graduate students participated in the research, and our study’s behavioral results indicated that reward (relative to no reward) and negative emotion (relative to positive and neutral emotion) significantly improved recognition performance. The ERP results showed that there were significant interactions between monetary reward and emotion on memory retrieval, and the reward effects of positive, neutral, and negative memory occurred at varied intervals in mean amplitude. The reward effect of positive memory appeared relatively early, at 260–330 ms after the stimulus onset in the frontal-frontocentral area, at 260–500 ms in the centroparietal-parietal area and at 500–700 ms in the frontocentral area. However, the reward effects of neutral and negative memory occurred relatively later, and that of negative memory appeared at 500–700 ms in the frontocentral and centroparietal area and that of neutral memory was at 500–700 ms in the frontocentral and centroparietal-parietal area. Meanwhile, significant FN400 old/new effects were observed in the negative and rewarded positive items, and the old/new effects of negative items appeared earlier at FN400 than positive items. Also, significant late positive component (LPC) old/new effects were found in the positive, negative, and rewarded neutral items. These results suggest that, monetary reward and negative emotion significantly improved recognition performance, and there was a mutual influence between reward and emotion on brain activity during memory retrieval.

## Introduction

Questions pertaining to memory retrieval attract a lot of attention because the ability to retrieve life experiences is crucial to our survival—for example, correctly recognizing a dangerous situation that we have encountered before can help us to prevent harm arising from that particular set of circumstances in the future. According to dual-process accounts, memory retrieval involves two distinct approaches: *familiarity* and *recollection* (Mandler, 1980; Jacoby and Dallas, 1981; Jacoby, 1991; Yonelinas, 2002; Rugg and Curran, 2007). Familiarity is the state in which people sense that an item is familiar but cannot recall any detailed information, whereas recollection is the condition in which people can not only correctly recognize learned “old” items but can also extract the details of the encoding episode. Event-related potential (ERP) studies of recognition memory have found that ERP waveforms elicited by correctly classified “old” items are more positive than waveforms elicited by correctly classified “new” items, which is known as the “old/new effect” in a recognition phase (Friedman and Johnson, 2000; Rugg and Curran, 2007). There are two types of common ERP old/new effects: that representing familiarity is reflected by an earlier frontal negative component (an FN400 that peaked at around 400 ms post-stimulus) while that representing recollection is reflected by a late positive component (LPC) peaking at about 600 ms with maximum amplitude at the parietal electrodes (Curran and Hancock, 2007; Rugg and Curran, 2007).

Several factors influence whether an event is remembered or forgotten, and emotions are one such factor. An “emotion” is defined as a physiological and behavioral response preceding a subjective experience that is marked by distinct bodily expression (James, 1884; Friedman, 2010). Emotions have widespread influences on human cognitive activities, and, specifically concerning the effect of emotion on memory, there are basically two types of research. One is focused on the effects of state-related mood on memory. For example, Kiefer et al. (2007) asked their study’s participants to first view funny or sad movies (i.e., inducing good and bad mood states) and then to memorize particular words. Their results showed a larger subsequent memory effect in a good rather than a bad mood at 500–650 ms of central electrodes. The other type of research investigates the effects of item-related emotion on memory, and has found that people are more likely to memorize emotional stimuli compared to neutral stimuli (Dolan, 2002; Kensinger and Corkin, 2003; LaBar and Cabeza, 2006; Grider and Malmberg, 2008). In addition, emotions mainly comprise two dimensions: valence and arousal. “Valence” refers to the extent of pleasure, varying from displeasure to pleasure, and “arousal” describes the intensity of the emotion, from calming to exciting (Russell, 1980; Lang et al., 1993). Previous researches have shown that valence and arousal both affect emotional memory, but they involve different neural processes and have some dissimilar impacts (Mickley Steinmetz and Kensinger, 2009; Xu et al., 2015). Our study focuses on the influence of item-related emotion on memory retrieval (and offers, below, a review of the relevant literature). Moreover, emotion in this study mainly concerns valence, and arousal was kept constant.

Some prior studies have linked memory enhancement to emotional stimuli (Dolan, 2002; Kensinger and Corkin, 2003; LaBar and Cabeza, 2006; Grider and Malmberg, 2008), and previous ERP studies have shown that emotional items enhance the amplitudes of LPC and/or FN400 compared to neutral items (Langeslag and van Strien, 2008; Weymar et al., 2009; Weymar et al., 2011). Moreover, negative events are usually retrieved on the basis of recollection, whereas positive and neutral events are recognized mainly on the basis of familiarity (Ochsner, 2000; Dolcos et al., 2004; Johansson et al., 2004; Kensinger and Corkin, 2004; Xu et al., 2015). However, other studies have found memory impairment in relation to emotional stimuli (Maratos et al., 2000; Corson and Verrier, 2007; Mackenzie et al., 2015) or a null effect (Dougal and Rotello, 2007; Kapucu et al., 2008). Thus, it remains unclear how emotions influence memory performance, and so the present study aims to investigate the role of emotion on, in particular, episodic memory.

Motivation is another factor that shapes memory. Numerous studies have used rewards to increase the motivational engagement of participants performing cognitive tasks, and have explored the effects of such motivation on memory performances. Most of these studies explore the effect of reward incentives induced during encoding on recognition memory, while a relatively few others examine the effect of the reward presented after the encoding on memory consolidation. For example, Nielson and Bryant (2005) found that extrinsic reward (monetary incentives) immediately after an encoding event improved retrieval performance in respect of a word list 1 week after the experiment, but intrinsic reward (praise) did not (both being compared with a control condition of no reward). Another comparatively small strand of this area of research investigates the effect of reward incentives presented upon the retrieval of a recognition memory. Several related studies have suggested that a reward at the point of retrieval speeds up participant reaction and boosts their confidence but does not improve the accuracy of these items, concluding that reward motivation at retrieval accelerates access to memory representations, but does not substantially change the quality of representations accessed (Bunzeck et al., 2009; Han et al., 2010; Marini et al., 2011). Relatedly, researchers have also suggested that the effect of a reward at retrieval on recognition can be affected by certain factors, such as the difficulty level of the memory tasks (Shigemune et al., 2017). In the present study, we focus on the effect of reward incentives induced during encoding on memory retrieval.

Previous studies from the behavioral perspective have explored the effects of reward anticipation during encoding on memory retrieval. Spaniol et al. (2013) investigated the influence of monetary reward anticipation on intentional episodic memory formation in younger and older adults, and found that both sets of participants showed enhanced recognition 24 h after the study for high-reward pictures compared with low-reward pictures, but the effect did not extend to immediate recognition following the study phase. Murayama and Kitagami (2014) observed that the reward cue facilitated an enhancement of retrieval for neutral pictures 1 week after the study, but not immediately upon testing. Oyarzún et al. (2016) asked participants to conduct a three-phase incidental encoding task, with one semantic category being rewarded in the second phase and a surprise recognition test being performed 24 h after the encoding. This study indicated recognition enhancement for inconsequential items only if the items were previously (but not subsequently) associated with a reward.

In addition to looking at the influence of reward on memory performance, prior studies have also explored the brain mechanisms of the reward effects on memory. On the one hand, functional magnetic resonance imaging (fMRI) studies have demonstrated that reward-induced memory enhancement involves a network of dopaminergic midbrain areas across the ventral striatum and hippocampus with increased dopamine release (Wittmann et al., 2005, 2008, 2013; Adcock et al., 2006; Bethus et al., 2010; Duzel et al., 2010; Shigemune et al., 2013). On the other hand, ERP investigations have highlighted temporal dynamics on brain activity corresponding to the effect (Eppinger et al., 2010; Marini et al., 2011; Halsband et al., 2012). For example, Eppinger et al. (2010) studied how reward-based learning affects ERP correlates of recognition memory in younger and older adults using a feedback-based learning task. They found that reward feedback learning, but not punishment feedback learning, improved recognition and brought about a significant early ERP old/new effect (250–400 ms) at the right and medial frontal regions, both in younger and older adults, which suggests that the reward feedback learning promoted a fast and automatic memory retrieval process. Marini et al. (2011) asked participants in their research to carry out an intentional memory task in respect of a neutral face, and set reward and no-reward conditions both in the study phase and within the test. Their behavioral data suggested that participants with a money reward were more accurate and faster in recognizing faces than those without reward, whether at the encoding or retrieval phase. The ERP results showed that there were greater subsequent memory effects for the rewarded than the unrewarded faces; beginning from 300 ms after the onset of the faces at study phase, and at retrieval, an early positive-going component for rewarded faces was found on the frontal regions, while the occipito-temporal N170 component showed priming effects of reward, and, on the frontocentral and centroparietal electrode clusters, the reward improved memory efficiency in the time windows of 300–500 ms and 500–700 ms.

It is well known that receiving rewards can evoke positive emotions, such as happiness and satisfaction, which in turn suggests that the reward has a close relationship with the resultant emotion. Plenty of empirical researches have also demonstrated this relation. For instance, in one study, reward circuits of the brain were shown to be activated when participants read stories about and imagined pleasant scenes (Costa et al., 2010). Emotionally positive stimuli such as smiling faces activate a similar neural network as a monetary reward incentive, including the striatum and its nucleus accumbens (Spreckelmeyer et al., 2009; Kohls et al., 2013; Rademacher et al., 2014), and the amygdala is activated both in the processing of emotional information and in receiving a reward (Baxter and Murray, 2002; Cardinal et al., 2002; Murray, 2007). These findings suggest a mutual influence between rewards and emotions.

In daily life, it is common and typical to seek happiness and benefit, and happy or reward-motivated events are more easily remembered. So far, however, there have only been a few studies that have investigated the joint effect of reward motivation and emotion on memory in the same experiment. Wittmann et al. (2008) used fMRI to study functional interactions between emotional valence and reward on incidental memory formation. The retrieval was in one to 2 days after the study, and the results showed that the reward improved recollection under positive emotional conditions but not under neutral or negative emotional conditions. Meanwhile, the neuroimaging results suggested positive stimuli promoted reward-related activity in the ventral striatum, but negative stimuli did not, which supported the suggestion that the ventral striatum plays a key role in the modulation of the reward output and memory formation in the hippocampus was influenced by positive emotional valence. In their study using positron emission tomography (PET), Shigemune et al. (2010) asked participants to perform an intentional memory task with regard to negative or neutral pictures with a high or low reward respectively. The retrieval test 24 h after the study showed that recognition performance was significantly enhanced by negative emotion (versus neutral emotion) and high monetary reward (rather than low monetary reward), but an interaction between emotion and reward was not discovered; the imaging result suggested that the right hippocampus may integrate the effects of emotion (processed in the amygdala) and the monetary reward (processed in the orbitofrontal cortex) on episodic memory encoding. Obviously, there are inconsistencies in the behavioral and neuroimaging results of the two studies, and it is necessary to more deeply explore the joint effect of reward motivation and emotion on memory formation, consolidation, and retrieval. Moreover, compared with fMRI and PET measurements, ERP indicators can better reflect a temporal stream of neural activity because of their high temporal resolution. Therefore, the present study used ERP measures to answer the intriguing question of whether monetary reward and emotion influence the same or different memory retrieval processes. More specifically, our study attempts to determine which ERP component—FN400 or LPC—might be influenced by monetary reward and emotion, and infer whether and how monetary reward and emotion affect familiarity or recollection processes. We hope to clarify the effect of monetary reward and emotion on memory retrieval and so gain insight into the relation between the two.

In the present study, recognition tests were conducted immediately after the encoding, using blocks as units. Participants were asked to conduct intentional memory tasks involving two factors: reward (comprising reward and no-reward conditions) and emotion (including positive, neutral, and negative emotion conditions). Based on previous findings, our prediction was that positive or negative emotion (compared with neutral emotion) and monetary reward (compared with no reward) would improve recognition performance, and that significant positive ERP average amplitude in old items with reward, rather than without reward, in positive pictures would appear relatively early, while, in respect of positive, neutral, and negative pictures, valence and reward would affect their corresponding FN400 old/new effects and the LPC old/new effects.

## Materials and Methods

### Participants

Twenty healthy adults participated in this study (10 of whom were male; all were between 20 and 28 years old, mean age = 23.15). All participants were right-handed undergraduate and graduate students from Capital Normal University in China and had normal or corrected-to-normal vision. This study was approved by the Institutional Review Board of the Capital Normal University, and all the methods were carried out in accordance with its relevant guidelines. All participants signed written informed consent prior to the experiments in accordance with the Declaration of Helsinki. No vulnerable populations were involved in this study. Following completion of the experiment, each participant received basic compensation as well as a monetary reward corresponding to their performance of the memory task in the study.

### Materials

The target stimuli consisted of 648 full-color images (216 positive, 216 negative, and 216 neutral) selected from the Chinese Affective Picture System (CAPS) (Lu et al., 2005). All images were uniform in size, through a few treatments. According to the CAPS, the emotional valence of the positive, neutral, and negative images respectively were 6.92 ± 0.43, 5.25 ± 0.48, and 2.70 ± 0.63, there being significant differences between the three types of pictures [*t*_(430)_ > 38.18, *p*s < 0.001]. There was no significant difference in emotional arousal between positive (5.51 ± 0.61) and negative pictures (5.57 ± 0.70) [*t*_(430)_ = -0.88, *p* > 0.05], but there were significant differences between positive or negative and neutral pictures (3.97 ± 0.57) [positive vs. neutral: *t*_(430)_ = 27.13, *p* < 0.001; negative vs. neutral: *t*_(430)_ = -26.07, *p* < 0.001]. Positive, negative, and neutral pictures were respectively divided into three groups, each group including 72 pictures, and the three groups were matched on emotion valence and arousal (see **Table 1**). Two groups of the matched three groups were randomly selected as target pictures during the study phase, for the reward and no reward conditions respectively. The other group, as the “new” items, together with the learned pictures (“old” items), was used in the test phase. To avoid primacy and recency effects, a further eight positive, eight negative, and eight neutral pictures from the CAPS were selected as filler stimuli. In addition, eight pictures each of positive, negative, and neutral emotion from the CAPS were selected as practice stimuli, but they did not appear in the formal experiments.

**Table 1:** The average valence and arousal of the matched three groups in positive, negative and neutral pictures.

	Emotion	Group1	Group2	Group3	*F*_(2,213)_	*p*	$\eta_{p}^{2}$
Valence	Positive	6.91 ± 0.05	6.93 ± 0.05	6.92 ± 0.05	0.02	0.981	<0.001
	Neutral	5.25 ± 0.06	5.26 ± 0.06	5.26 ± 0.06	0.01	0.991	<0.001
	Negative	2.72 ± 0.08	2.71 ± 0.08	2.68 ± 0.08	0.07	0.930	0.001
Arousal	Positive	5.51 ± 0.07	5.52 ± 0.07	5.50 ± 0.07	0.02	0.982	<0.001
	Neutral	3.97 ± 0.07	3.96 ± 0.07	3.97 ± 0.07	<0.01	0.996	<0.001
	Negative	5.57 ± 0.08	5.56 ± 0.08	5.57 ± 0.08	<0.01	0.999	<0.001

*The data after “±” in the table are the standard errors of the mean.*

### Procedures

Participants were told that they would take part in a memory experiment in which they would receive some monetary reward if they remembered the pictures preceded by reward cues in the study phase later. The reward rules for the task were that participants were given a basic compensation (RMB 55; namely, USD 8.19), and would obtain a further RMB 0.20 (USD 0.03) for each correct recognition of old items in the test phase, but would lose RMB 0.15 (USD 0.02) for each false alarm (an “old” response to a “new” item). Participants were paid total monetary compensation (basic plus rewards) in cash after the experiment. Before the experiment, each participant performed a training exercise to familiarize themselves with the requirements of the study.

**FIGURE 1:**
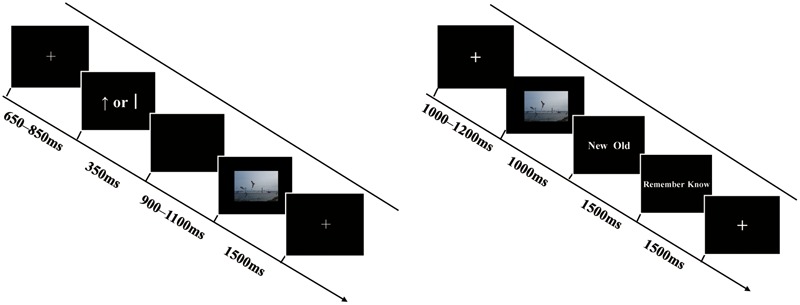
Schematic representations of a trial in the study phase (the left) and retrieval phase (the right). See text for details. The example picture resembles those in the experiment but it is not part of the CAPS.

The formal experiment comprised six blocks, each containing a study, a distraction, and retrieval phase (see **Figure 1**). In the study phase, each trial began with a cross fixation point presented for 650–850 ms. Following the fixation, a reward cue (“”) or no-reward cue (“j”)—based on Shigemune et al. (2013)—was displayed on the screen for 350 ms, with the symbols “” (indicating a monetary reward associated with the upcoming picture) and “j” (indicating no reward for the upcoming picture) displayed equally in terms of length, thickness, and occurrence frequency. Then, the cue was replaced by a blank screen of 900–1100 ms. After that, a picture was presented for 1500 ms and participants were asked to memorize the picture (and, meanwhile, confirm that they were human through pressing a key), and then the next trial began. In total, participants studied 72 target pictures (24 positive, 24 neutral, and 24 negative) and 4 filler pictures (2 filler pictures were at the beginning and 2 filler pictures at the end of the sequence) in the study phase of each block. Participants’ performances regarding filler pictures were not analyzed. After the study, participants were immediately asked to carry out a distraction task concerning subtraction beginning with a three-digit number (i.e., repeatedly subtracting 3 from each number) for 30 s, then to complete a retrieval test after a rest for 2 min. In the retrieval phase of each block, participants performed 108 trials, comprising 72 trials for old pictures and 36 trials for new pictures (12 positive, 12 neutral, and 12 negative). In each test trial, a cross fixation was first presented for 1,000–1,200 ms, followed by a picture for 1,000 ms. Next, the text “new/old” was displayed on the screen for 1,500 ms and participants were asked to perform an old/new judgment and indicate their response by pressing “F” or “J” on the keyboard within the 1,500 ms. If they judged the present picture as an old one, then they were required to proceed with the “remember/know” judgment for 1,500 ms by pressing “C” or “M” on the keyboard. Participants were instructed to respond “remember” when they believed that a picture had been presented in the study and they were able to recollect details associated with the picture. They were asked to respond “know” when they were sure that the picture had been presented in the study, but they could not recollect any details, only a feeling of it being familiar. Participants were asked to make quick and accurate judgments. The order of the trials was pseudo-random and successive; three or more trials with the same emotional valence did not occur in trial sequences in the study and retrieval tasks. The order of the six blocks was counterbalanced across participants. The assignment of the left and right hand to all response buttons also was counterbalanced across participants.

### ERP Recordings and Analysis

Electroencephalographic (EEG) data were recorded by a 62-channel Neuroscan system (Neuroscan SynAmps2; NeuroScan Inc., Sterling, VA, United States) at 500 Hz sampling rate with a 0.05–100 Hz bandpass filter. The electrode locations conform to the extended international 10–20 system. Electrooculogram (EOG) was recorded with two pairs of electrodes, one pair placed above and below the left eye and another pair at the outer canthi of both eyes. All electrodes were referenced on-line to the left mastoid and re-referenced off-line to the average of the right and left mastoid recordings. EOG blink artifacts were corrected using a linear regression estimate (Picton et al., 2000; Hornberger et al., 2004; Wang et al., 2015). Impedances were kept below 5 kΩ. EEG and EOG signals were band-pass filtered from 0.05 to 40 Hz. Trials with a voltage exceeding ± 75 μV were excluded from the ERP analysis. Each average epoch lasted 1,200 ms, including 200 ms prior to picture onset served as a baseline.

According to related ERP literature (Marini et al., 2011) and visual inspection of the grand average ERP waveform, the two electrode clusters were selected, incorporating frontal-frontocentral electrodes (F3, Fz, F4, FC3, FCz, FC4) and centroparietal-parietal electrodes (CP3, CPz, CP4, P3, Pz, P4), and the time windows of 260–330 ms, 330–500 ms, and 500–700 ms were designated. Average amplitudes over left, midline, and right electrodes in each of the four brain locations (frontal, frontocentral, centroparietal, and parietal) were calculated because pilot analyses didn’t show an obvious influence of laterality on the joint effect of reward and emotion on memory retrieval. Our analyses focused on reward effects, emotion effects, and their interaction in respect of old items, as well as old/new effects under each condition. To analyze reward and emotion effects regarding old items, 2 (reward type: reward vs. no reward) × 3 (emotion: positive, neutral, and negative) × 2 (location: frontal vs. frontocentral, or centroparietal vs. parietal) repeated-measures analyses of variance (ANOVAs) were conducted on the average amplitudes of each time window respectively for frontal-frontocentral and centroparietal-parietal locations. To analyze old/new effects, 2 (item type: rewarded old vs. new, or non-rewarded old vs. new) × 2 (location: frontal vs. frontocentral, or centroparietal vs. parietal) repeated-measures ANOVAs respectively under positive, neutral, and negative condition were conducted on average amplitudes for each time window. Repeated-measures ANOVAs were corrected using the Greenhouse–Geisser method (Greenhouse and Geisser, 1959). The alpha level was 0.05. Multiple comparisons or simple effect analyses were corrected using the Bonferroni correction. All data analyses were conducted using SPSS statistics software.

Because fewer than 16 items per condition were judged as “know” items by most participants (75%), ERP analyses of the remember/know judgment were not carried out.

## Results

### Behavioral Data

#### Hit Rates, False Alarm Rates, and Memory Discrimination Accuracies in Old/New Judgments

Participants’ recognition performances in the old/new judgments are given in **Table 2**. The Shapiro–Wilk tests for normality showed that the data under each condition were normally distributed (*W*s > 0.91, *p*s > 0.05). For the ANOVA analysis, outliers with a Cook’s distance > 1 (Cook and Weisberg, 1982) were detected also on the data, and no outliers were found, showing that these significant findings were not statistical artifact.

**Table 2:** Mean Hit rates (HRs), False alarm rates (FARs), and Prs in each condition of old/new judgment.

	Reward			No reward		
	Positive	Neutral	Negative	Positive	Neutral	Negative
HRs	0.83 ± 0.03	0.77 ± 0.04	0.79 ± 0.03	0.78 ± 0.03	0.73 ± 0.04	0.78 ± 0.04
FARs	0.18 ± 0.02	0.13 ± 0.02	0.10 ± 0.01	0.18 ± 0.02	0.13 ± 0.15	0.10 ± 0.01
Prs	0.65 ± 0.03	0.64 ± 0.04	0.69 ± 0.03	0.60 ± 0.03	0.59 ± 0.04	0.67 ± 0.04

*The data after “ ± ” in the table are the standard errors of the mean.*

With hit rates (i.e., the percentage of “old” responses to old items) as the dependent variable, a two-way repeated-measures ANOVA was performed with reward type (rewarded old, non-rewarded old) and emotion (positive, neutral, negative) as factors. The results showed that the main effects of reward type and emotion were both significant [*F*_(1,19)_ = 17.15, *p* = 0.001, $\eta_{p}^{2}$ = 0.47; *F*_(2,38)_ = 5.41, *p* < 0.05, $\eta_{p}^{2}$ = 0.22] without significant interaction between the two factors [*F*_(2,38)_ = 1.93, *p* > 0.05, $\eta_{p}^{2}$ = 0.09]. Further analysis showed that reward (compared to no reward) and positive emotion (compared to neutral emotion) significantly enhanced hit rates (*p* = 0.001 and *p* < 0.05).

A one-way repeated-measures ANOVA on false alarm rates (i.e., the percentage of “old” responses to new items) with emotion (positive, neutral, negative) as the factor suggested a significant main effect of emotion [*F*_(2,38)_ = 17.66, *p* < 0.001, $\eta_{p}^{2}$ = 0.48]. The multiple comparisons showed that the average false alarm rate for positive items was higher than for neutral and negative items (*p*s < 0.01), and the average for neutral items was higher than that for negative items (*p* < 0.05). Thus, participants had a response bias for positive new items given “old” responses, but had the lowest false alarm rate for negative items.

Following Snodgrass and Corwin (1988), accuracy was classified using the memory discrimination accuracy index Pr, which was calculated by the hit rate minus the false alarm rate. Prs were tested using a two-way repeated-measures ANOVA with reward type (reward, no reward) and emotion (positive, neutral, negative) as factors. The results indicated significant main effects of reward type and emotion [*F*_(1,19)_ = 17.15, *p* = 0.001, $\eta_{p}^{2}$ = 0.47; *F*_(2,38)_= 6.92, *p* < 0.01, $\eta_{p}^{2}$ = 0.27] without significant interaction between the two factors [*F*_(2,38)_ = 1.93, *p* > 0.05, $\eta_{p}^{2}$ = 0.09]. Further analysis suggested the Pr of old items with reward was significantly higher than those without reward (*p* = 0.001), and the Pr of negative items was significantly higher than that of positive and neutral items (*p*s < 0.01), suggesting that reward (compared to no reward) and negative emotion (compared to positive or neutral emotion) significantly improved overall recognition accuracy.

#### Memory Discrimination Accuracies in Remember/Know Judgments

The Prs of remember/know judgments under different emotional conditions were calculated by means of the hit rates minus the false alarm rates (Snodgrass and Corwin, 1988), as shown in **Table 3**. They were analyzed using a three-factor repeated measures ANOVA with reward type (reward, no reward), emotion (positive, neutral, negative), and remember/know responses as factors. The results showed that all the main effects of reward, emotion, and the remember/know response were significant [*F*_(1,19)_= 17.15, *p* = 0.001, $\eta_{p}^{2}$ = 0.47; *F*_(2,38)_ = 6.92, *p* < 0.01, $\eta_{p}^{2}$ = 0.27; *F*_(1,19)_ = 24.31, *p* < 0.001, $\eta_{p}^{2}$ = 0.56], and the interactions between reward and the remember/know response and between emotion and the remember/know response were also significant [*F*_(1,19)_ = 5.60, *p* < 0.05, $\eta_{p}^{2}$ = 0.23; *F*_(2,38)_ = 8.53, *p* = 0.001, $\eta_{p}^{2}$ = 0.31]. A simple effect analysis indicated that the Pr of the “remember” response to old items with reward was significantly higher than that for old items without reward (*p* < 0.01), and the ones for positive and negative items were significantly higher than for neutral items (*p*s < 0.001); meanwhile, the Pr of the “know” response to positive items was significantly lower than that to negative or neutral items (*p*s < 0.05).

**Table 3:** Mean Prs of remember and know responses in each condition.

R/K judgement		Reward			No reward		
		Positive	Neutral	Negative	Positive	Neutral	Negative
Remember	Prs	0.56 ± 0.05	0.50 ± 0.05	0.57 ± 0.05	0.51 ± 0.05	0.44 ± 0.05	0.54 ± 0.05
Know	Prs	0.09 ± 0.04	0.14 ± 0.05	0.12 ± 0.04	0.09 ± 0.04	0.15 ± 0.04	0.13 ± 0.04

*The data after “ ± ” in the table are the standard errors of the mean.*

### ERP Data

**FIGURE 2:**
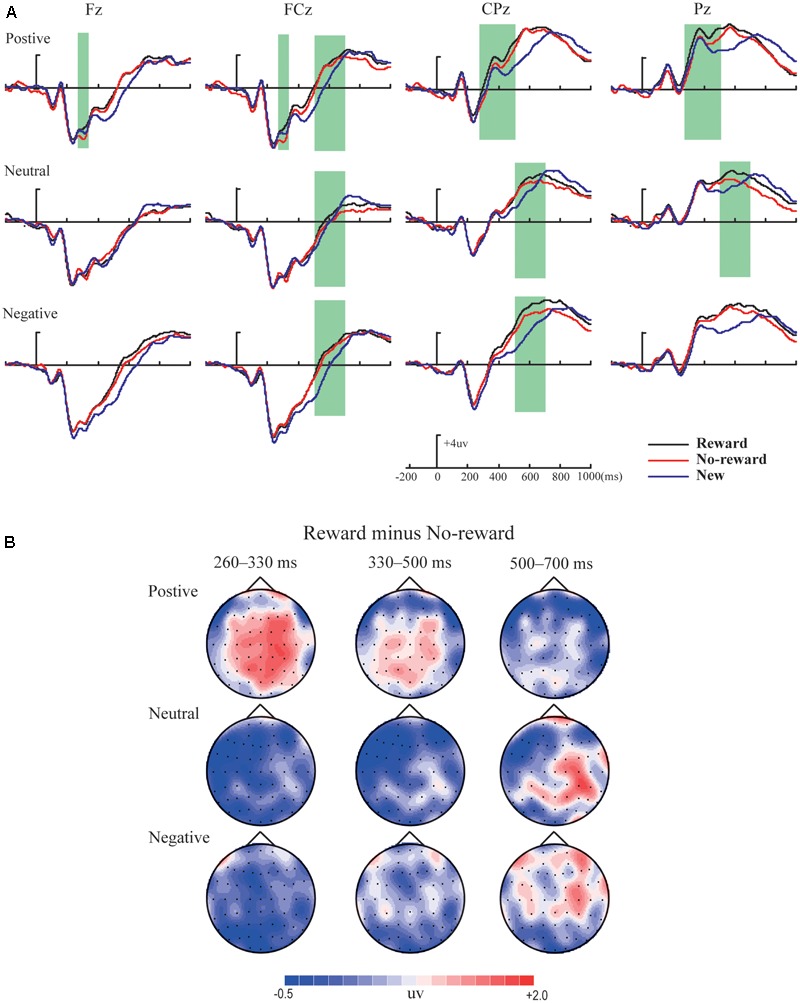
The amplitude distribution and topographic map of ERP about reward effects in positive, neutral and negative emotion conditions. **(A)** The amplitude comparison of old items with reward, old items without reward and new items under three emotional conditions. **(B)** The topographic map of ERP about reward effects (reward minus no-reward) under three emotional conditions.

**FIGURE 3:**
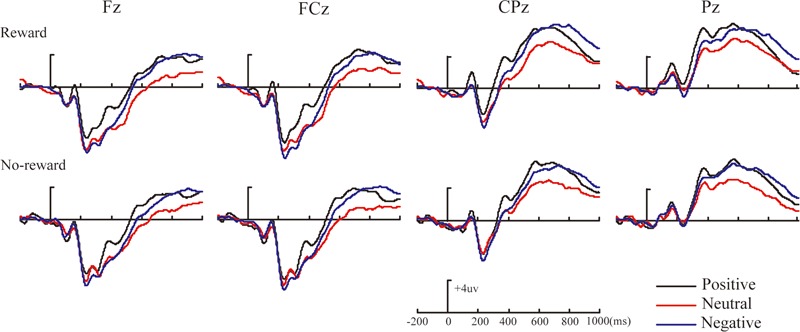
The amplitude distribution of ERP about emotion effects in reward and no-reward items at Fz, FCz, CPz, and Pz.

**FIGURE 4:**
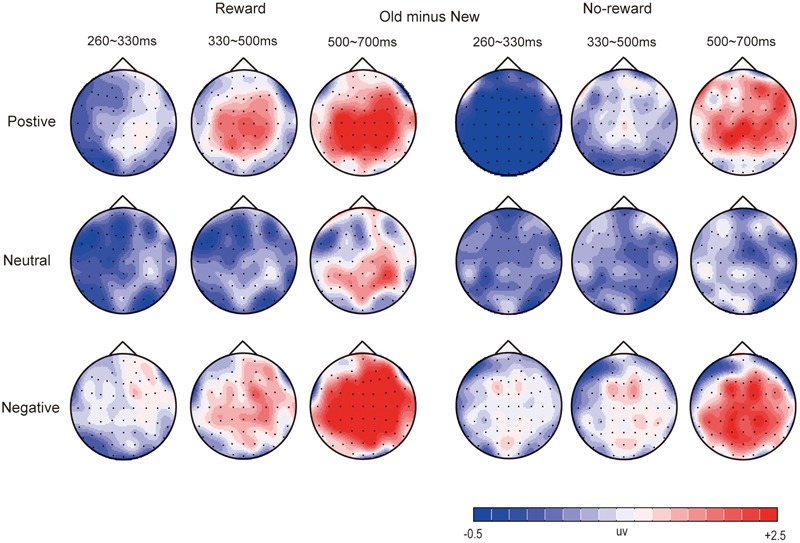
The topographic map of ERP about old/new effects in rewarded and no-rewarded old items under positive, neutral, and negative conditions.

**Figures 2–4** illustrate the ERP results of the present study. The amplitude distribution and topographic map of ERP measurements in relation to reward effects in positive, neutral, and negative emotion conditions are shown in **Figure 2**. The amplitude distributions of ERP around emotion effects under reward and no-reward conditions are illustrated in **Figure 3**. Moreover, **Figure 4** shows the topographic maps of old/new effects under reward and no-reward conditions of positive, neutral, and negative items.

### Reward and Emotion Effects in Old Items

#### Frontal-Frontocentral Area

##### Time window 260–330 ms

The ANOVA revealed a significant main effect of emotion [*F*_(2,38)_ = 10.87, *p* < 0.001, $\eta_{p}^{2}$ = 0.36] and a marginally significant interaction between reward type and emotion [*F*_(2,38)_ = 3.26, *p* = 0.053, $\eta_{p}^{2}$ = 0.15]. Simple effect analysis showed that significantly more positive average amplitude in old items with reward than without reward (i.e., reward effect) was found only under the positive condition (*p* < 0.05), and, for emotion effects, only in old items with reward, the average amplitude of positive pictures was significantly more positive than for neutral and negative pictures (*p*s < 0.05).

##### Time window 330–500 ms

Variance analysis indicated a significant main effect of emotion [*F*_(2,38)_ = 18.85, *p* < 0.001, $\eta_{p}^{2}$ = 0.50], and multiple comparisons showed significantly more positive average amplitude for positive pictures than neutral and negative pictures (*p*s < 0.001).

##### Time window 500–700 ms

The ANOVA revealed a significant main effect of emotion [*F*_(2,38)_ = 14.02, *p* < 0.001, $\eta_{p}^{2}$ = 0.43], and multiple comparisons showed significantly more positive average amplitude of positive and negative pictures than neutral pictures (*p*s < 0.05). Additionally, a significant interaction between reward type and brain location [*F*_(1,19)_ = 5.30, *p* < 0.05, $\eta_{p}^{2}$ = 0.22] was obtained, and simple effect analysis showed significant reward effect only in the frontocentral area (*p* < 0.05).

#### Centroparietal-Parietal Area

##### Time window 260–330 ms

Variance analysis showed significant main effects of reward type and emotion [*F*_(1,19)_ = 4.77, *p* < 0.05, $\eta_{p}^{2}$ = 0.20; *F*_(2,38)_ = 6.11, *p* < 0.01, $\eta_{p}^{2}$ = 0.24]. The interaction between reward type and emotion was also significant [*F*_(2,38)_ = 4.40, *p* < 0.05, $\eta_{p}^{2}$ = 0.19], and simple effect analysis revealed that the significant reward effect appeared only under the positive condition (*p* < 0.05), and significantly more positive average amplitude for positive pictures than neutral and negative pictures only in old items with reward (*p*s < 0.05). Also, a significant interaction between emotion and location was found [*F*_(2,38)_ = 6.52, *p* < 0.01, $\eta_{p}^{2}$ = 0.26], and simple effect analysis showed significantly more positive average amplitude for positive pictures than neutral and negative pictures both in the centroparietal and parietal areas (*p*s < 0.05).

##### Time window 330–500 ms

The ANOVA revealed significant main effects of reward type and emotion [*F*_(1,19)_ = 9.87, *p* < 0.01, $\eta_{p}^{2}$ = 0.34; *F*_(2,38)_ = 11.31, *p* < 0.001, $\eta_{p}^{2}$ = 0.37] and a significant interaction between emotion and location [*F*_(2,38)_ = 10.72, *p* < 0.001, $\eta_{p}^{2}$ = 0.36]. Simple effect analysis showed significantly more positive average amplitude for positive pictures than neutral pictures in the centroparietal area (*p* < 0.001) and significantly more positive average amplitude for positive and negative pictures than neutral pictures in the parietal area (*p*s < 0.05). Although no significant interaction between reward type and emotion [*F*_(2,38)_ = 1.08, *p* > 0.05, $\eta_{p}^{2}$ = 0.05] was found, based on careful inspection of amplitude distribution and topographic maps of ERP (see **Figure 2**), we performed further planned comparisons (Keppel, 1991), which incorporated 2 (reward type: reward vs. no reward) × 2 (location: centroparietal vs. parietal) repeated-measures ANOVAs respectively under the positive, neutral, and negative conditions. These results revealed that reward effect was significant under the positive condition [*F*_(1,19)_ = 7.32, *p* < 0.05, $\eta_{p}^{2}$ = 0.28], but was not significant under the neutral and negative conditions [*F*_(1,19)_ = 1.16, *p* > 0.05, $\eta_{p}^{2}$ = 0.06; *F*_(1,19)_ = 1.53, *p* > 0.05, $\eta_{p}^{2}$ = 0.08].

##### Time window 500–700 ms

There were significant main effects of reward type and emotion [*F*_(1,19)_ = 9.74, *p* < 0.01, $\eta_{p}^{2}$ = 0.34; *F*_(2,38)_ = 13.11, *p* < 0.001, $\eta_{p}^{2}$ = 0.41]. The interaction among reward type, emotion, and location was also significant [*F*_(2,38)_ = 3.60, *p* < 0.05, $\eta_{p}^{2}$ = 0.16], and simple effect analysis revealed the significant reward effect of neutral pictures in the centroparietal-parietal area (*p* < 0.05) and the significant reward effect of negative pictures only in the centroparietal area (*p* < 0.05). Additionally, significantly more positive average amplitudes of positive and negative pictures than neutral pictures were found (*p*s < 0.05).

#### Summary

Within our ERP data, the reward effect of positive memory appeared at 260–330 ms after the stimulus onset in the frontal-frontocentral area, at 260–500 ms in the centroparietal-parietal area, and at 500–700 ms in the frontocentral area, while the reward effect of neutral pictures emerged at 500–700 ms in the frontocentral and centroparietal-parietal areas and that of negative pictures appeared at 500–700 ms in the frontocentral and centroparietal areas (see **Figure 2**). Thus, the reward effect of the positive pictures occurred earlier than for the neutral and negative pictures. Meanwhile, regarding emotion effects, the data suggested that the mean amplitudes for positive pictures were significantly more positive than for neutral or negative items, at 260–700 ms, except that there was no significant difference between positive, neutral, and negative pictures without reward at 260–330 ms; the mean amplitudes of negative pictures became significantly more positive than those for neutral items at 330–500 ms in the parietal area, and at 500–700 ms in the frontal-frontocentral and centroparietal-parietal areas (see **Figure 3**).

### Old/New Effects

#### Frontal-Frontocentral Area

Under the reward condition, for positive pictures, significant old/new effects (i.e., significantly more positive average amplitude for correctly recognized old pictures than for correctly rejected new pictures) were found at 330–500 ms and 500–700 ms [*F*_(1,19)_ = 22.89, *p* < 0.001, $\eta_{p}^{2}$ = 0.55; *F*_(1,19)_ = 24.55, *p* < 0.001, $\eta_{p}^{2}$ = 0.54]. For negative pictures, the significant old/new effects appeared at 260–330 ms, 330–500 ms, and 500–700 ms [*F*_(1,19)_ = 6.30, *p* < 0.05, $\eta_{p}^{2}$ = 0.25; *F*_(1,19)_ = 14.23, *p* < 0.01, $\eta_{p}^{2}$ = 0.43; *F*_(1,19)_ = 38.47, *p* < 0.001, $\eta_{p}^{2}$ = 0.67].

Under the no-reward condition, the old/new effect of positive pictures was significant only at 500–700 ms [*F*_(1,19)_ = 14.33, *p* < 0.001, $\eta_{p}^{2}$ = 0.43]. The old/new effects of negative pictures were significant at 260–330 ms, 330–500 ms, and 500–700 ms [*F*_(1,19)_ = 9.35, *p* < 0.01, $\eta_{p}^{2}$ = 0.33; *F*_(1,19)_ = 28.77, *p* < 0.001, $\eta_{p}^{2}$ = 0.60; *F*_(1,19)_ = 28.50, *p* < 0.001, $\eta_{p}^{2}$ = 0.60].

#### Centroparietal-Parietal Area

Under the reward condition, for positive pictures, significant old/new effects were found at 330–500 ms and 500–700 ms [*F*_(1,19)_ = 27.59, *p* < 0.001, $\eta_{p}^{2}$ = 0.59; *F*_(1,19)_ = 27.79, *p* < 0.001, $\eta_{p}^{2}$ = 0.59]. For neutral pictures, the old/new effect was significant at 500–700 ms [*F*_(1,19)_ = 11.36, *p* < 0.01, $\eta_{p}^{2}$ = 0.37]. For negative pictures, a significant interaction between item type and location was found at 260–330 ms [*F*_(1,19)_ = 4.67, *p* < 0.05, $\eta_{p}^{2}$ = 0.20], and simple effect analysis showed a significant old/new effect only in the centroparietal area; significant old/new effects also emerged at 330–500 ms and 500–700 ms [*F*_(1,19)_ = 18.22, *p* < 0.001, $\eta_{p}^{2}$ = 0.49; *F*_(1,19)_ = 51.94, *p* < 0.001, $\eta_{p}^{2}$ = 0.73].

Under the no-reward condition, a significant old/new effect for positive pictures was found only at 500–700 ms [*F*_(1,19)_ = 18.66, *p* < 0.001, $\eta_{p}^{2}$ = 0.50]. Significant old/new effects for negative pictures were found at 260–330 ms, 330–500 ms, and 500–700 ms [*F*_(1,19)_ = 8.29, *p* < 0.01, $\eta_{p}^{2}$ = 0.30; *F*_(1,19)_ = 15.03, *p* < 0.001, $\eta_{p}^{2}$ = 0.44; *F*_(1,19)_ = 58.00, *p* < 0.001, $\eta_{p}^{2}$ = 0.75].

#### Summary

Significant old/new effects for negative pictures emerged the earliest, starting at 260 ms regardless of old items with reward or no-reward, while those for positive pictures secondly began at 330 ms (and only in the old items with reward), and significant old/new effects of neutral pictures were found only at 500–700 ms in the centroparietal-parietal area, and only between the old items with reward and new items (see **Figures 2, 4**).

## Discussion

Our study explored the effects of monetary reward and emotion on episodic memory using ERP, mainly investigating the retrieval phase. Notably, we found there was a mutual influence of money reward and emotion on memory retrieval, and that the reward effects of positive, neutral, and negative emotional memory occurred at different time intervals in the ERP mean amplitude. The reward effects of positive items appeared relatively early, at 260–330 ms after stimulus onset from frontal to parietal areas, at 330–500 ms in the centroparietal-parietal area and at 500–700 ms in the frontocentral area. However, the reward effects of neutral and negative items occurred relatively later: that for neutral pictures emerged at 500–700 ms in the frontocentral or centroparietal-parietal areas and that for negative pictures appeared at 500–700 ms in the frontocentral or centroparietal areas. These results indicate that the time dynamic and affected brain area for the reward effects in relation to positive, neutral, and negative items were different. A detailed discussion of our results follows.

### Behaviors

At the behavioral level, although we found reward (compared to no reward) and positive emotion (compared to neutral emotion) significantly enhanced hit rates, we also found that emotion significantly influenced the false alarm rates of new items, and participants had response biases for positive new items given “old” responses, but had the lowest false alarm rate for negative items. So, based on Snodgrass and Corwin (1988), memory discrimination accuracies (Prs) were calculated according to hit rates minus false alarm rates. A statistical analysis of the Prs showed significant main effects of reward and emotion without a significant interaction of the two factors. Specifically, reward (compared to no reward) significantly improved recognition performance, which replicates reward effects shown in previous memory studies (Wittmann et al., 2008; Eppinger et al., 2010; Marini et al., 2011; Halsband et al., 2012; Shigemune et al., 2013). The reward effects in our study might be related to the reward anticipation during the encoding prompting a motivated learning, which ensured an efficient allocation of cognitive resources (Marini et al., 2011). That is, more cognitive resources were allotted to the items with a reward, but not to the items without a reward. Meanwhile, we also found that negative emotion significantly enhanced Prs compared to positive and neutral emotions, which is again consistent with prior studies (Kensinger et al., 2007; Schaefer et al., 2011; Mao et al., 2015). In line with our results, Shigemune et al. (2010) found that negative emotion (rather than neutral emotion) and high monetary reward (rather than low monetary reward) improved Prs without an interaction of the two, but their study did not include positive stimulus. In contrast, Wittmann et al. (2008) used fMRI to study functional interactions between emotional valence and reward on incidental memory formation, and found that reward enhanced recollection (Prs) under positive conditions but not under neutral or negative conditions, which is different from the results of the present study. Moreover, in the studies of Wittmann et al. (2008) and Shigemune et al. (2010), the memory tests were applied 24 h after encoding, and the memory task in Wittmann et al. (2008) was incidental while that in Shigemune et al. (2010) was intentional, and, therefore, we can’t determine the reason that inconsistent results were obtained. Further investigation needs to be carried out.

In addition, in remember/know judgments, the Prs of “remember” responses to old items with reward (vs. those with old items without reward) and negative old items (vs. those with positive and neutral items) was significantly higher. Thus, participants showed more recollection on the old items with reward (compared to old items without reward) or negative old items (compared to positive and neutral items).

### ERPs

#### Interaction between Reward and Emotion on the FN400

Some prior ERP studies have suggested that the FN400 old/new effect at about 300–500 ms after the stimulus onset, mainly in the frontal area, is related to implicit memory processes based on familiarity (Curran and Hancock, 2007; Rugg and Curran, 2007), with “familiarity” referring to the idea that participants can only correctly recognize the learned old items, but cannot extract any details of the items (Yonelinas, 2002). In respect of the memory of positive pictures, at the 330–500 ms interval, the present study found significant old/new effects between old items with reward and new items, whether at the frontal-frontocentral area or the centroparietal-parietal area, but no significant old/new effect between the old items without reward and new items was observed, which suggests the reward in the study phase promoted familiarity with regard to positive pictures.

At 260–330 ms after the onset of positive pictures, although there was no significant old/new effect between the old items with or without reward and new items, a significant reward effect appeared in the frontal to parietal areas; moreover, significant reward effects in respect of the positive pictures also appeared at 330–500 ms in the centroparietal-parietal area and at 500–700 ms in frontocentral area. Previous studies have also suggested that a motivational stimulus, such as one with positive valence or high reward, would be the first to capture attention (Mogg et al., 2003; Kiss et al., 2009). We further infer that the early reward effect for positive pictures in the present study is probably connected to a joint effect between reward and emotion, which might capture more attention, evoke in-depth memory processing and faster recognition. Furthermore, in general, getting a reward is often associated with positive emotions such as happiness and satisfaction, and previous studies have shown that the amygdala in the processing of both emotional information and a reward is activated, suggesting a mutual influence between rewards and emotion (Baxter and Murray, 2002; Cardinal et al., 2002; Murray, 2007). Wittmann et al.’s (2008) findings that a reward improved recollection only under positive emotional conditions and positive stimuli promoted reward-related activity in the ventral striatum supports the suppositions that the ventral striatum plays a key role in the modulation and the reward system and memory formation in the hippocampus is influenced by positive emotional valence. Based on these previous results, we posit that the mutual influence between rewards and positive emotion may facilitate the earlier occurrence of the reward effect in respect of positive pictures in the present study. Because of the mutual influence between rewards and positive emotion and co-activation in related brain areas, we also expected to observe an enhancement in memory of positive items, compared to that for neutral and negative pictures, as Wittmann et al. (2008) found, but we did not discover this result. One possible reason for this is that a surprise retrieval test was given 24 h after the main experiment in Wittmann et al. (2008), whereas, in our study, an immediate test after the study in the block design was applied.

In the present study, an enhancement was observed in the Prs in respect of negative pictures rather than for positive and neutral pictures. The ERP data showed, regardless of whether with or without reward, the FN400 old/new effect of negative pictures emerged the earliest, starting at 260 ms; the FN400 old/new effect of positive pictures between only the old items with reward and new items began next, at 330 ms; and that for neutral pictures was not found, whether at the frontal-frontocentral area or the centroparietal-parietal areas. It can be inferred that participants had obvious advantages in recognizing the negative pictures, consistent with the conclusions of previous studies (Kensinger et al., 2007; Schaefer et al., 2011; Mao et al., 2015). Moreover, the participants also had obvious advantages in recognizing positive pictures over neutral pictures. Because the FN400 old/new effect indicates familiarity (Curran and Hancock, 2007; Rugg and Curran, 2007), the above results, together with the data of remember/know judgments showing the Prs of “know” responses to negative items were significantly higher than positive items, affirm that negative emotion enhances familiarity regarding old items whether with reward or without (the reward had little impact on familiarity in respect of the negative pictures). Our ERP data also suggest that reward promoted familiarity only with regard to positive items with reward, but not for negative or neutral pictures.

#### Interaction between Reward and Emotion on the LPC

Previous studies have elucidated that the LPC old/new effect suggests recollection (Curran and Hancock, 2007; Rugg and Curran, 2007). Additionally, a number of related ERP studies have revealed that LPC old/new effects are enhanced in positive and negative items, compared to neutral items, in the retrieval phase (Langeslag and van Strien, 2008; Weymar et al., 2009, 2010, 2011), while other studies have shown that negative emotion improved the LPC old/new effect, compared with positive and neutral emotion (Kensinger et al., 2007; Schaefer et al., 2011; Mao et al., 2015). In the current study, significant LPC old/new effects were found at 500–700 ms in the positive, negative, and rewarded neutral items, which indicates that positive or negative emotion facilitates recollection in old items and reward promotes recollection of neutral items.

Similar to the results of Marini et al. (2011), our study revealed a significant LPC reward effect between neutral items with reward and without reward at 500–700 ms. Marini et al. (2011) also found the reward effect in relation to a neutral stimulus in 300–500 ms at the frontocentral area, which was not found in the current study. In addition to a significant reward effect in respect of neutral items, the present study showed a significant LPC reward effect in respect of negative items. Halsband et al. (2012) used ERP to investigate the effects of high and low reward on retrieval orientation of object words, and found the reward-associated retrieval orientation effect at 400–700 ms and 700–1000 ms after the stimulus onset in the frontal area. But, in the present study, the significant reward effects in regard to neutral pictures was mainly at the frontocentral or centroparietal-parietal areas, the significant reward effect for negative memory appeared in the frontocentral or centroparietal areas, and that for positive memory was at 260–500 ms also in the centroparietal-parietal area and at 500–700 ms at the frontocentral areas. Related studies have shown that reward or motivational relevance can facilitate memory performance by way of dopaminergic projections from the amygdala and the ventral tegmental area to the hippocampus and the striatum (Lisman and Grace, 2005; Brombergmartin et al., 2010; Miendlarzewska et al., 2015), and our results suggest that the LPC reward effect in respect of negative and neutral items might be relevant to this mechanism. Although we found a difference of reward effects between positive, neutral, and negative emotions in occurrence time and brain area, further investigation concerning the inconsistency and its deep brain mechanism is needed.

In addition, it should be noted that, in the present study, although we found that negative valence enhanced memory performance, we also found a more positive ERP for positive pictures than for neutral or negative pictures in most time windows. Koenig and Mecklinger (2008) investigated the impact of emotional content on encoding and retrieval, and also found that positive events yielded anterior and posterior slow-wave activity, compared to neutral and negative events. This slow wave for positive items suggests more attention resources are invoked for cognitive processing. Some studies have found that positive emotion will give rise to attention expansion and more cognitive flexibility, and individuals under positive emotion tend toward up–down or integral information processing (Rowe et al., 2007; Koenig and Mecklinger, 2008), but also will omit some concrete details (Gasper and Clore, 2002), which possibly brings about a familiar feeling in response to positive pictures and thus a high response bias toward positive new items given “old” responses, evoking significantly high hit rates of positive (rather than neutral) old items.

On the basis of the findings above, it can be seen that the results in this study support those of prior related studies on the influence of reward motivation and emotion on episodic memory, and also extend previous findings by demonstrating that the brain activity of the reward effect is affected by emotion, and that the reward effect in respect of positive items appears relatively early while those of negative or neutral items occur relatively later. These results will help to facilitate further in-depth discussion on neural mechanisms with reference to the effects of motivation and emotion on episodic memory.

Furthermore, our findings have implications for improving students’ memory performance. Students often encode emotional information in real life as when they recite a poem eliciting pleasant feeling, or when they try to memorize related information of a tragic event. Meanwhile, in order to encourage good memory performance, parents or teachers sometimes promise the students some rewards. Then, how do emotion and reward anticipation influence students’ memory? The current study gave a preliminary answer. First, reward motivation during learning can improve subsequent recognition. Therefore, in real life, it might be useful to provide a prize or a gift for enhancing a student’s memory efficiency. Second, negative emotion can significantly prompt recognition performance, suggesting one can better remember dangerous situation closely related to survival. However, for positive and neutral information, students might need more effort to memorize them. Third, the present study showed that positive information with reward during encoding would be retrieved faster, which reveals a joint effect of monetary reward and emotion on episodic memory retrieval. However, we also found a high response bias toward positive new items given “old” responses. Therefore, in real life students should notice that they possibly give a false recognition for a positive item because of the familiar feeling in response to positive items.

There are also several limitations in the present study. First, only the two reward types (reward, no reward) were manipulated in this research, and the monetary reward of only RMB 0.20 (0.03 USD) for each correct recognition was relatively small. Future studies should examine the effects of different levels of monetary reward (e.g., high reward, low reward, no reward) on episodic memory retrieval. Secondly, we used a relatively small sample size. Although our experiment attained sufficient powers to detect the effect of reward motivation and emotion on memory retrieval, according to the effect size and *p*-values, similar studies with larger sample sizes may be required to further support our findings. Third, the results in this study were obtained from college students and should be studied in relation to other participant groups in order to establish their generalizability.

## Conclusion

The current study used ERPs to investigate the cognitive and brain mechanisms of monetary reward and emotion affecting the retrieval processes of episodic memory. The behavioral results indicated that a reward (relative to no reward) and a negative emotion (relative to positive and neutral emotion) significantly improved recognition performance, with no interaction between the two factors. Our ERP results, though, revealed significant interactions between the reward and the emotion, and the reward effects of a positive, neutral, or negative emotional memory occurred at varied intervals. The reward effect of the positive emotional memory appeared relatively early, at 260–330 ms after stimulus onset in the frontal-frontocentral area, and at 260–500 ms in the centroparietal-parietal area and at 500–700 ms in frontocentral area. However, the reward effects of the neutral or the negative emotional memory occurred relatively later, and, furthermore, that of the negative memory appeared at 500–700 ms in the frontocentral and centroparietal area and that of the neutral memory emerged at 500–700 ms in the frontocentral and centroparietal-parietal area. Meanwhile, significant FN400 old/new effects were observed in respect of the negative and rewarded positive items, and the old/new effects of the negative items appeared earlier at FN400 than those of the positive items. Significant LPC old/new effects were found in the positive, negative, and rewarded neutral items. Together, these results suggest that a monetary reward and a negative emotion can significantly improve recognition performance, and that there is a mutual influence between reward and emotion on brain activity during memory retrieval.

## Author Contributions

QZ supervised the project. CY designed the experiment, wrote the main manuscript text and prepared **Figures 1–4** and **Tables 1–3**. CY, FL, and YL collected and analyzed the experimental data. Besides, QZ, LC, and CY reviewed the manuscript.

## Conflict of Interest Statement

The authors declare that the research was conducted in the absence of any commercial or financial relationships that could be construed as a potential conflict of interest.
